# Supplementary material for: Ocean dynamics shape marine heatwaves and their predictability
Source: Nat Commun. 2026 Feb 18;17:2896. doi: 10.1038/s41467-026-69509-7 (PMC13031658; doi:10.1038/s41467-026-69509-7)
Supplement: Supplementary file 1 — Supplementary Information [file 41467_2026_69509_MOESM1_ESM.pdf]

Supplementary Information for

Ocean dynamics shape marine heatwaves and their predictability

Xianglin Ren[1], Wei Liu[1,*], Liping Zhang[2,3]

[1]Department of Earth Sciences and Planetary Sciences, University of California Riverside, Riverside, CA, USA.

[2]NOAA/Geophysical Fluid Dynamics Laboratory, Princeton, NJ, USA

[3]University Corporation for Atmospheric Research, Boulder, CO, USA

*Corresponding author. Email: wei.liu@ucr.edu

This file includes:

Supplementary Figures 1-12 and Table 1

Supplementary Figures

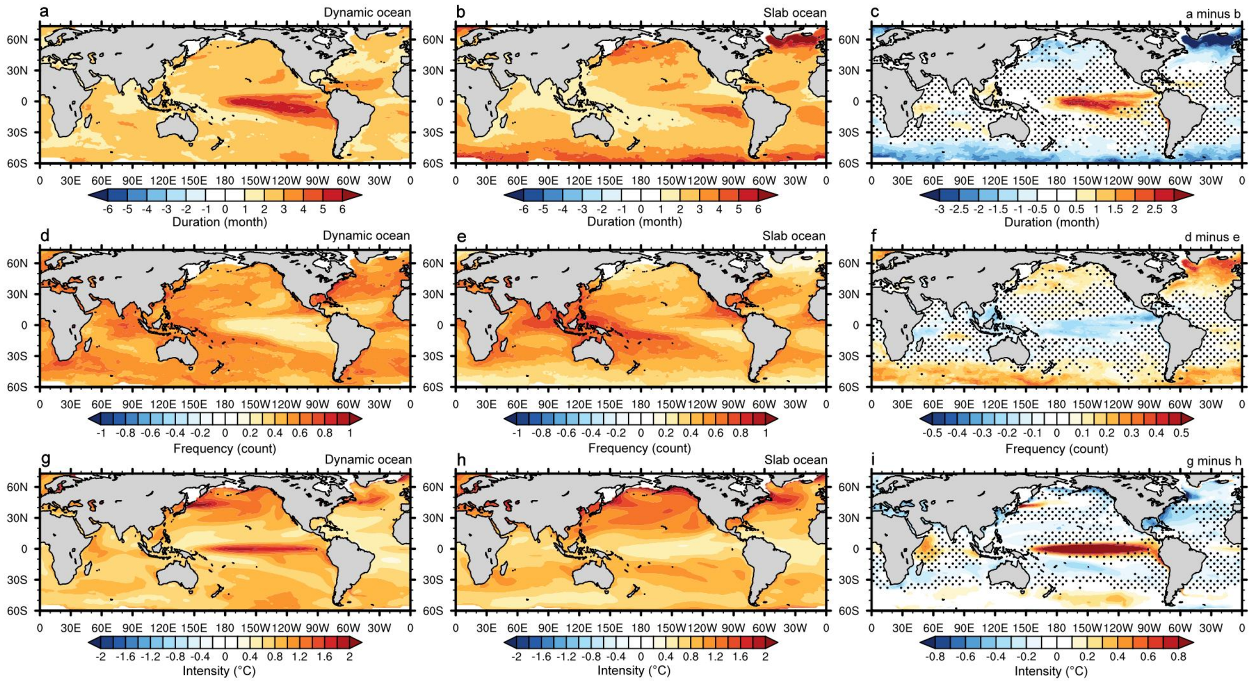

Supplementary Fig. 1 | Marine heatwaves in dynamic and slab oceans based on a monthly definition. (a-c) Marine heatwave durations (color shading in months) of (**a**) dynamic and (**b**) slab oceans, as well as (**c**) the difference between the two (dynamic minus slab). **(d–f)** Same as (**a–c**) but for marine heatwave annual frequencies. **(g–i)** Same as (**a–c**) but for marine heatwave intensities. Different from Fig. 1, marine heatwaves here are based on the monthly marine heatwave definition (Methods). The stipples in panels (**c**, **f**, **i**) refer to the regions where differences are statistically insignificant based on Student's t-test at the 95% confidence level. The base map is from NCAR Command Language map outline databases.

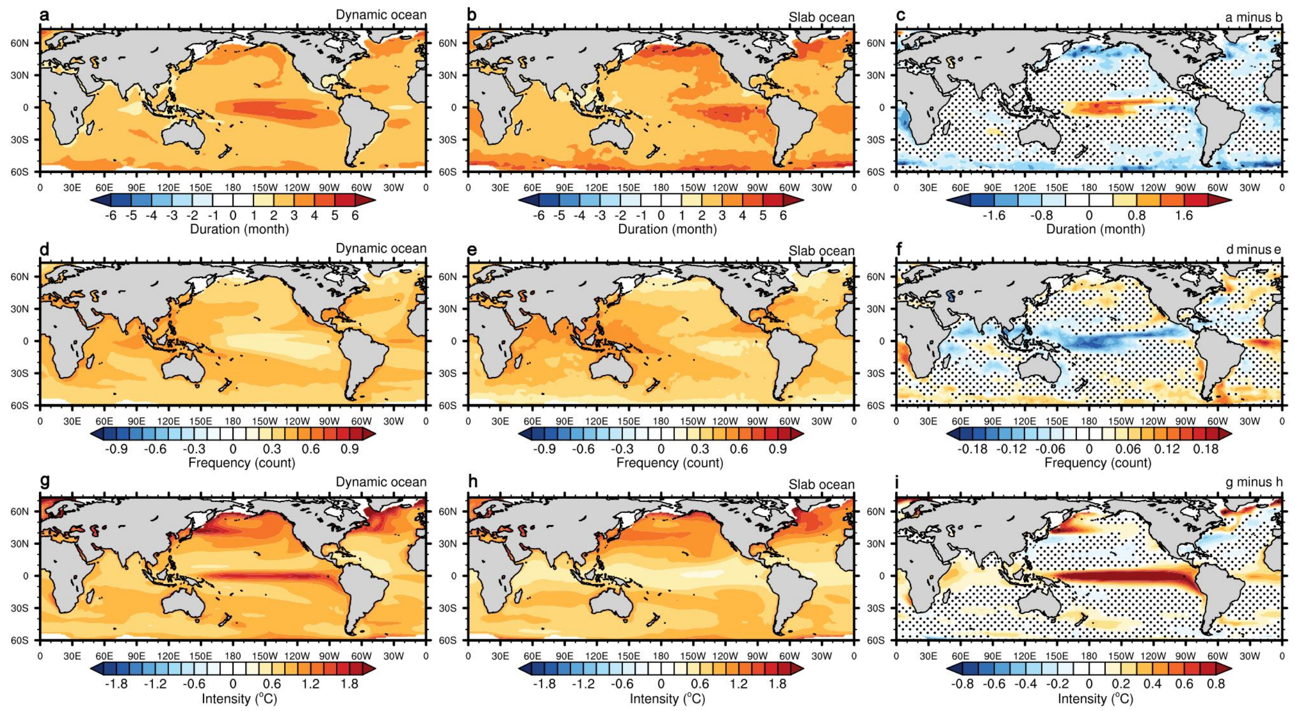

Supplementary Fig. 2 | Marine heatwaves in dynamic and slab oceans of CMIP3 models.

(**a-c**) Marine heatwave durations (color shading in months) of (a) dynamic and (b) slab oceans, as well as (c) the difference between the two (dynamic minus slab) for the multi-model mean of CMIP3 preindustrial simulations. (**d–f**) Same as (a–c) but for marine heatwave annual frequencies. (**g–i**) Same as (a–c) but for marine heatwave intensities. Different from Fig. 1, marine heatwaves here are based on the monthly marine heatwave definition (Methods). The stipples in panels (c, f, i) refer to the regions where differences are not statistically insignificant based on Student's t-test at the 95% confidence level. The base map is from NCAR Command Language map outline databases.

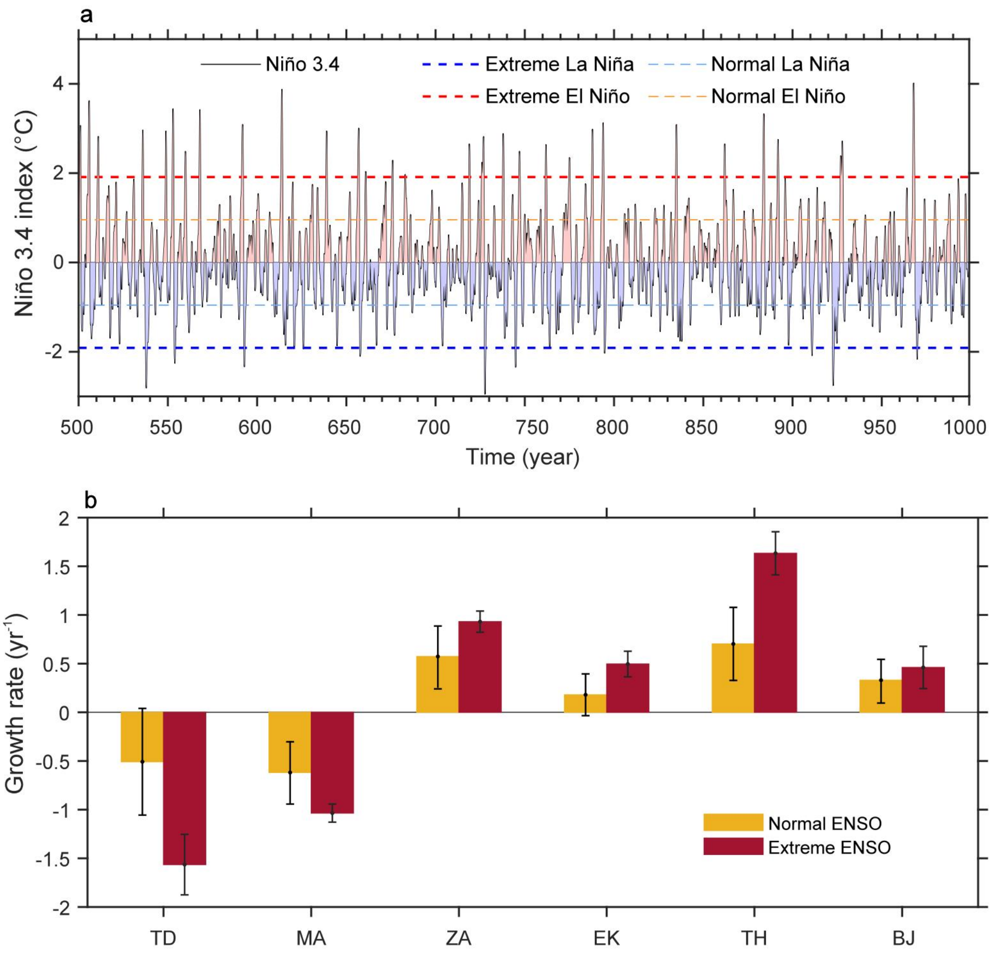

Supplementary Fig. 3 | Bjerknes stability for normal and extreme El Niño-Southern Oscillation.

(**a**) Niño 3.4 index from the dynamic ocean simulation, in which extreme El Niño-Southern Oscillation events are defined as sea surface temperature anomalies exceeding ±2σ (beyond the red and blue lines), and normal El Niño-Southern Oscillation events are defined as sea surface temperature anomalies from ±1σ to ±2σ (between the orange and light blue lines) where σ denotes one standard deviation of sea surface temperature. (**b**) The Bjerkness (BJ) index and individual components (Methods) of normal El Niño-Southern Oscillation (orange) and extreme El Niño-Southern Oscillation (red). TD, MA, ZA, EK, and TH represent the thermal damping, mean advection, zonal advection, Ekman upwelling, and thermocline feedbacks, respectively. Error bars denote one standard deviation of feedback term among El Niño-Southern Oscillation events.

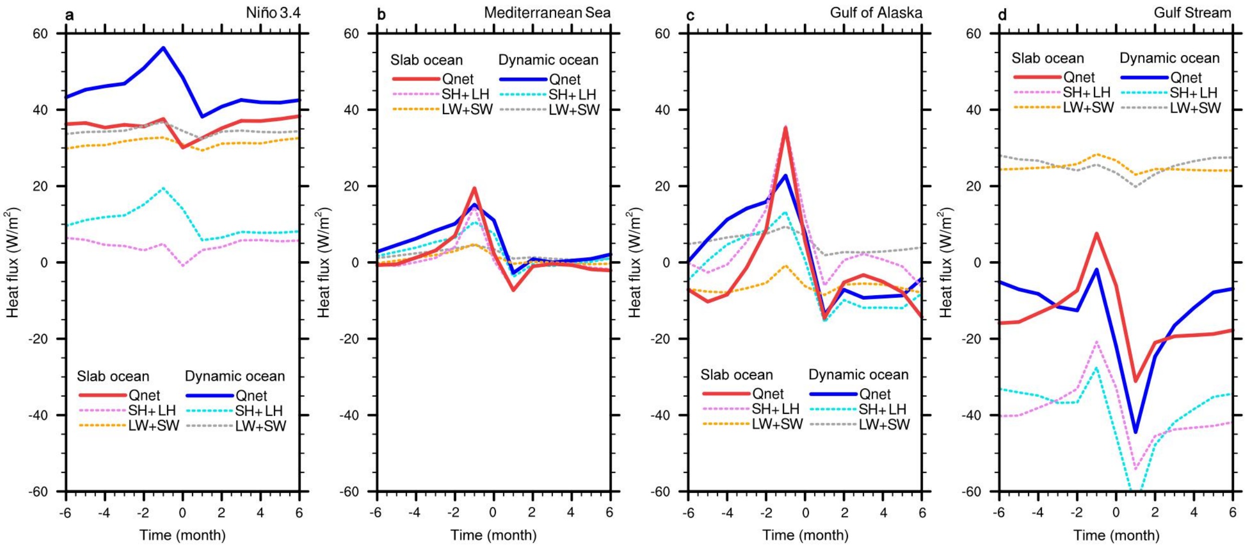

Supplementary Fig. 4 | Surface heat flux evolution during marine heatwave events. Evolution of regionally averaged surface heat flux components composited relative to marine heatwave peak (month 0) in four selected regions: the (**a**) Niño 3.4, (**b**) Mediterranean Sea, (**c**) Gulf of Alaska, and (**d**) Gulf Stream regions. Solid lines represent net surface heat fluxes (Qnet) for dynamic (blue) and slab (red) oceans. Dotted lines represent the components of net surface heat flux: the sum of longwave and shortwave radiative energy fluxes (LW+SW, dynamic ocean, gray; slab ocean, orange), and the sum of sensible and latent heat fluxes (SH+LH, dynamic ocean, light blue; slab ocean, magenta). Positive values indicate downward flux into the ocean.

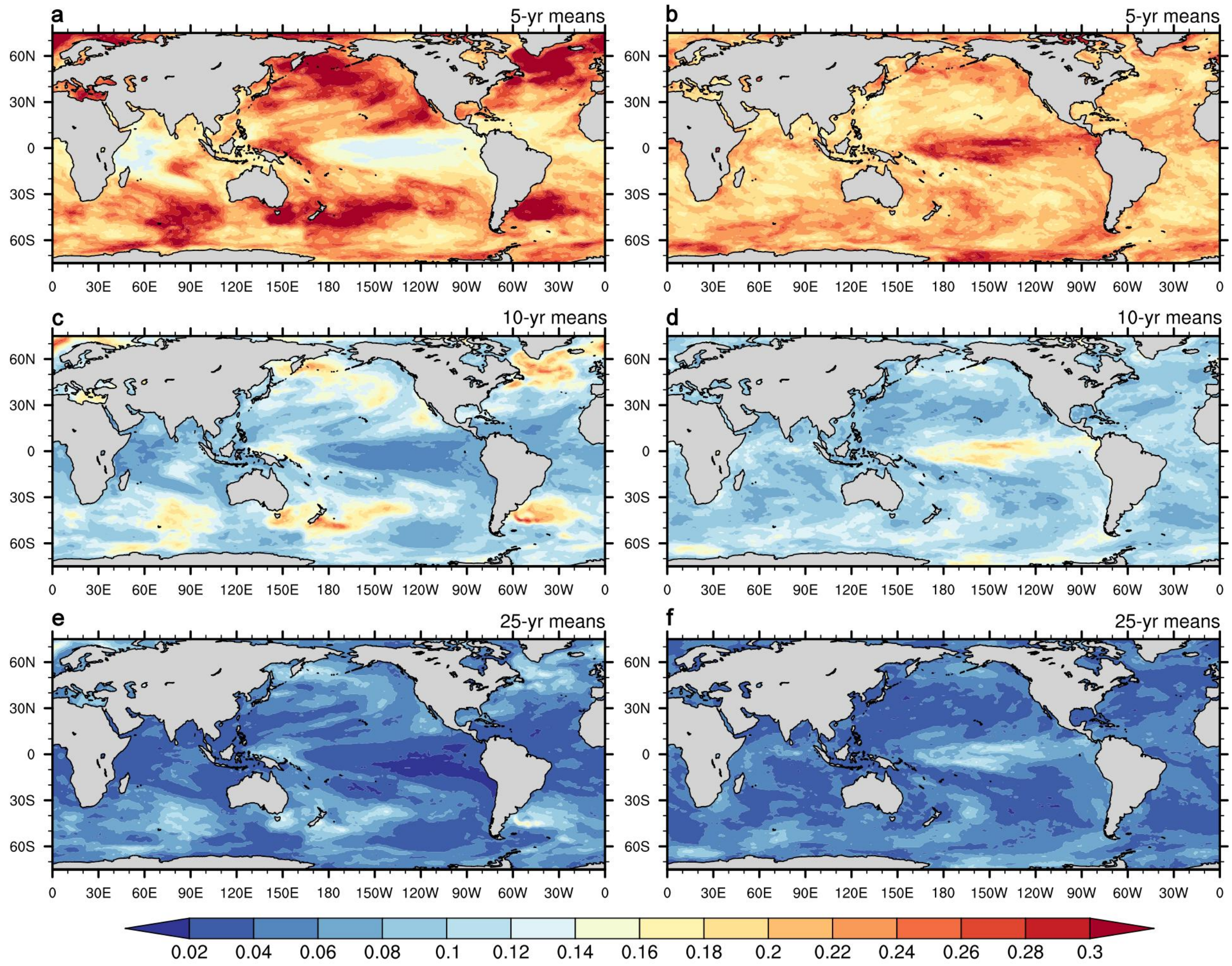

Supplementary Fig. 5 | Potential predictability variance fraction for marine heatwave duration. (**a, c, e**) Potential predictability variance fraction (ppvf) for (a) 5-year, (c) 10-year, and (e) 25-year average marine heatwave duration in dynamic ocean. (**b, d, f**) Same as (a, c, e) but for slab ocean. The base map is from NCAR Command Language map outline databases.

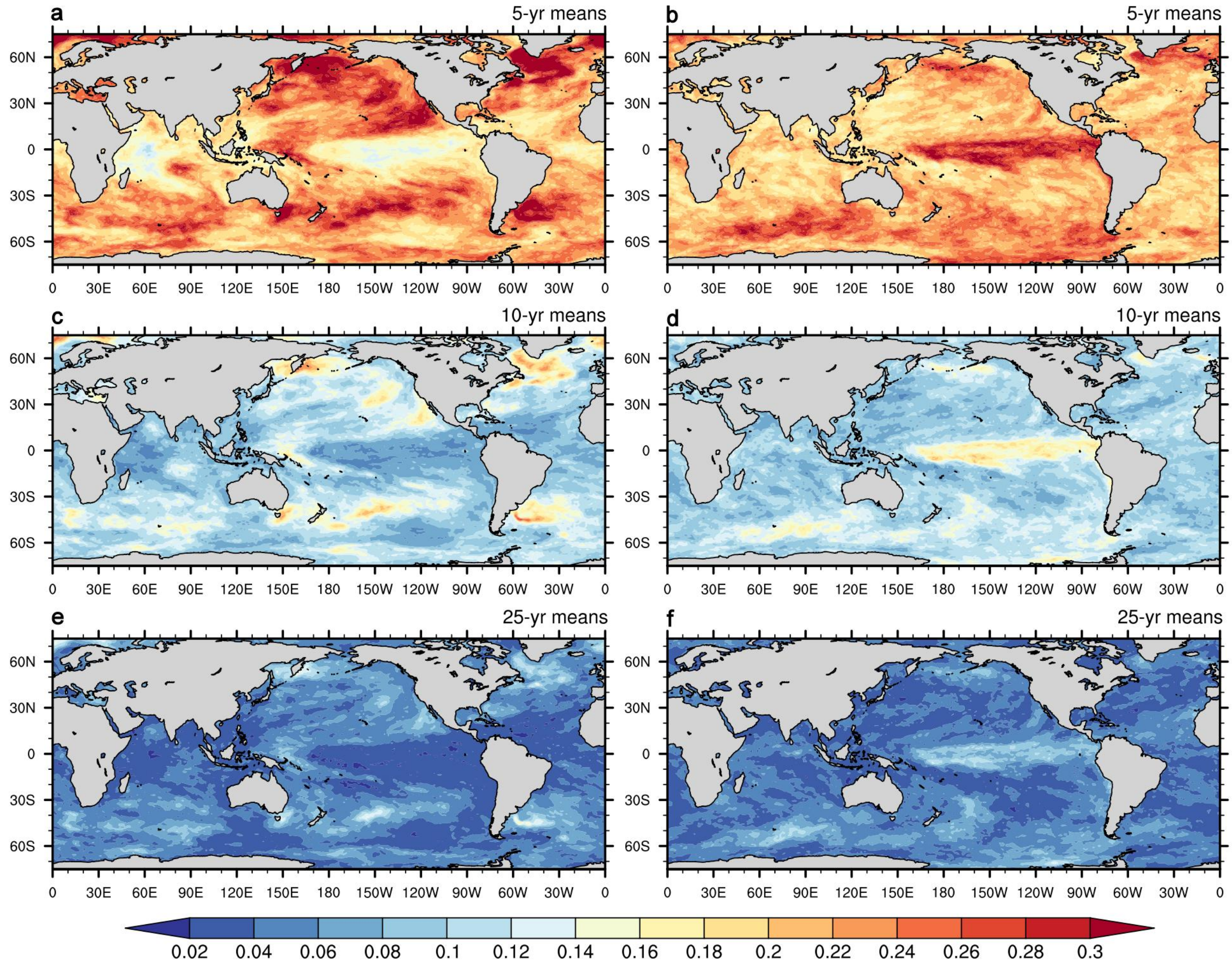

Supplementary Fig. 6 | Potential predictability variance fraction for marine heatwave intensity. (**a, c, e**) Potential predictability variance fraction (ppvf) for (a) 5-year, (c) 10-year, and (e) 25-year average marine heatwave intensity in dynamic ocean. (**b, d, f**) Same as (a, c, e) but for slab ocean. The base map is from NCAR Command Language map outline databases.

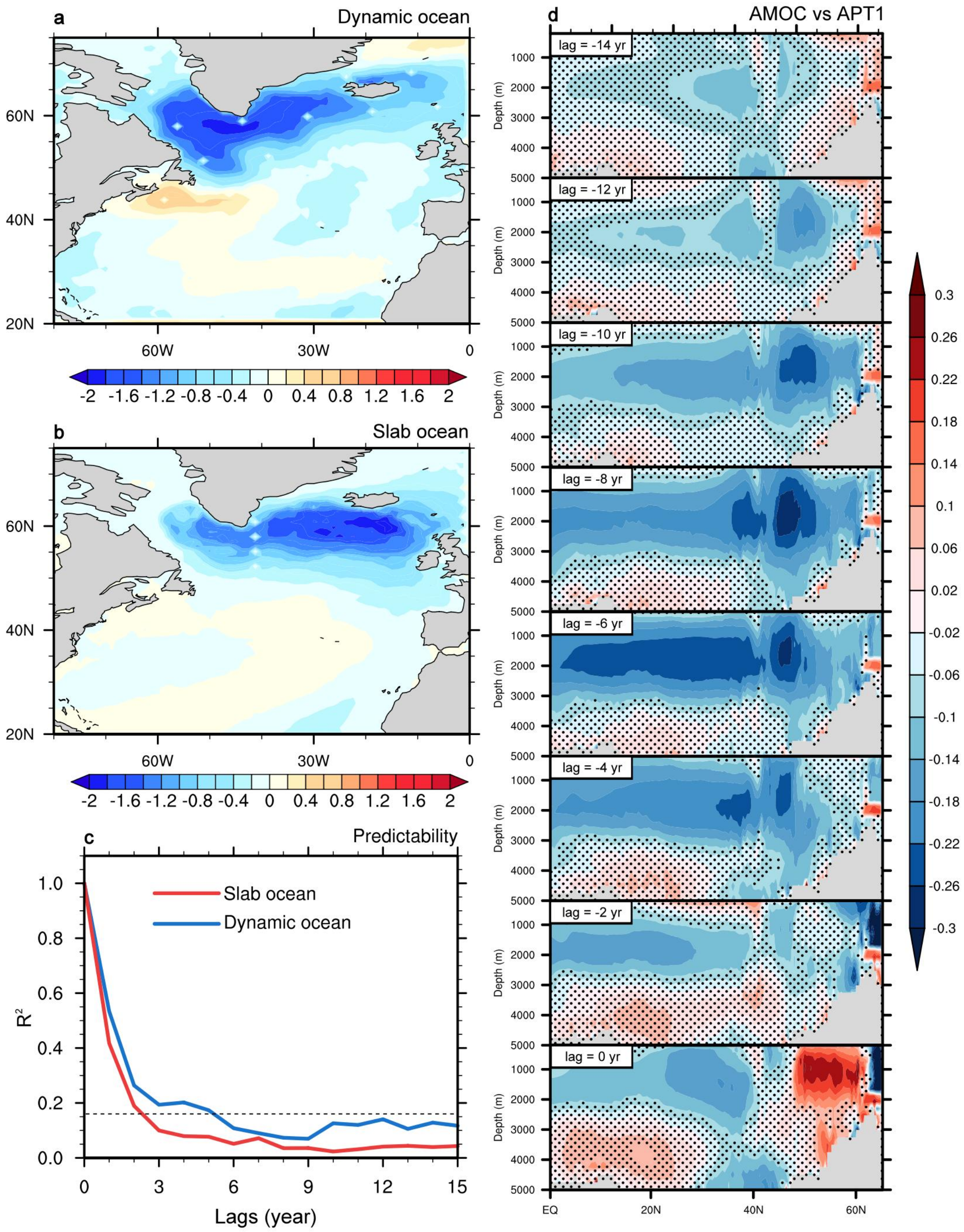

Supplementary Fig. 7 | North Atlantic marine heatwave duration predictability in dynamic and slab oceans. (**a-c**) The leading predictable component (APT1) of North Atlantic marine heatwave duration in (a) dynamic and (b) slab oceans, as well as (c) the squared multiple correlation coefficient R^2 (dynamic ocean, blue; slab ocean, red). The dashed black line in (c) denotes the 95% significance level. (**d**) Lead/lag correlation between Atlantic meridional overturning stream-function and APT1 index for North Atlantic marine heatwave duration. Lags range from –14 to 0 years, in which negative lags indicate that the Atlantic Meridional Overturning Circulation (AMOC) leads the APT index. The stipples refer to the regions where correlations are statistically insignificant at the 95% confidence level. The base map is from NCAR Command Language map outline databases.

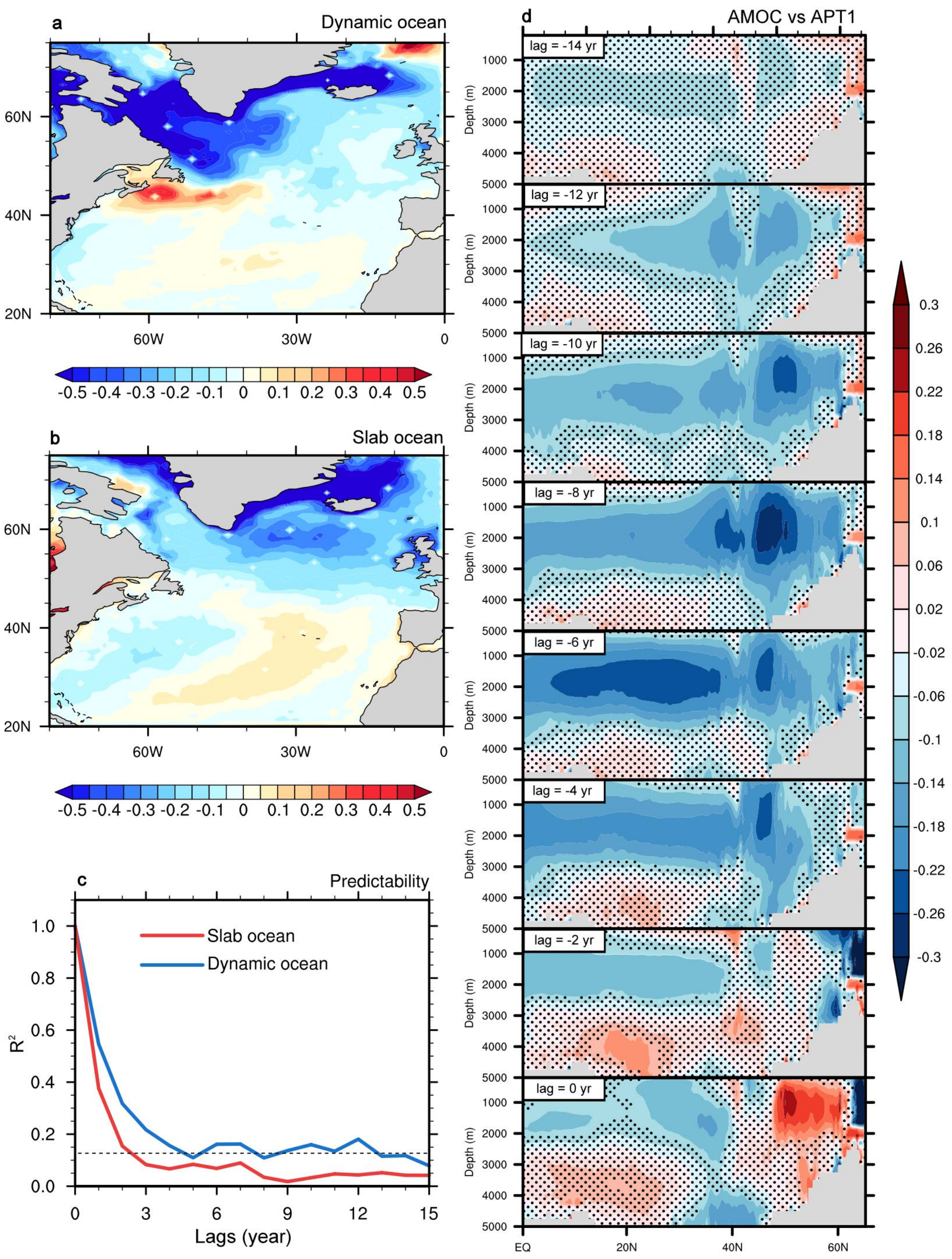

Supplementary Fig. 8 | North Atlantic marine heatwave intensity predictability in dynamic and slab oceans. (**a-c**) The leading predictable component (APT1) of North Atlantic marine heatwave intensity in (a) dynamic and (b) slab oceans, as well as (c) the squared multiple correlation coefficient R^2 (dynamic ocean, blue; slab ocean, red). The dashed black line in (c) denotes the 95% significance level. (**d**) Lead/lag correlation between Atlantic meridional overturning stream-function and APT1 index for North Atlantic marine heatwave intensity. Lags range from –14 to 0 years, in which negative lags indicate that the Atlantic Meridional Overturning Circulation (AMOC) leads the APT index. The stipples refer to the regions where correlations are statistically insignificant at the 95% confidence level. The base map is from NCAR Command Language map outline databases.

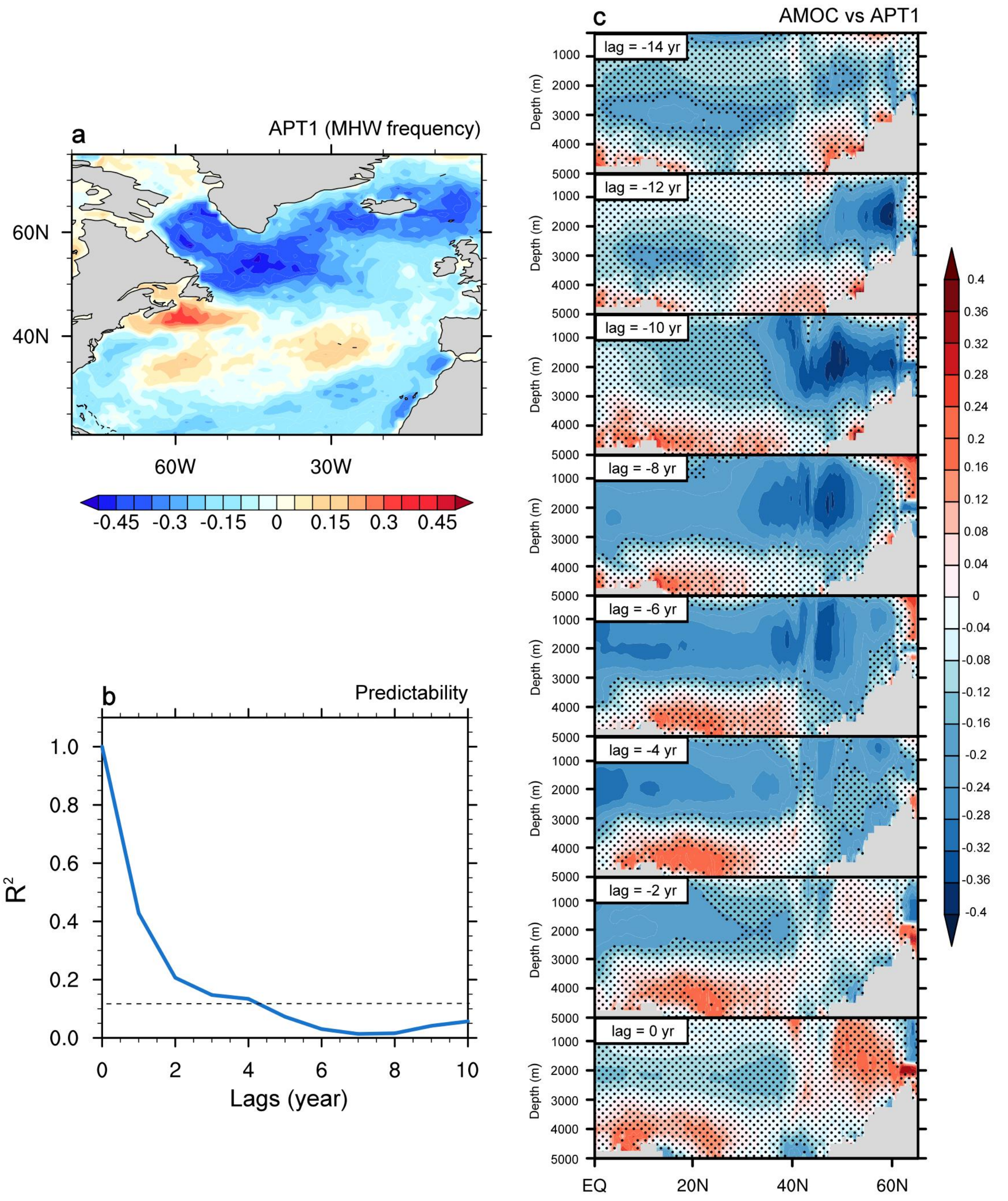

Supplementary Fig. 9 | North Atlantic marine heatwave frequency predictability in CESM1 historical simulations. (**a**) The leading predictable component (APT1) of North Atlantic marine heatwave annual frequency in CESM1 historical large ensemble simulations (Methods), and (**b**) the squared multiple correlation coefficient R^2. The dashed black line in (b) denotes the 95% significance level. (**c**) Lead/lag correlation between Atlantic meridional overturning stream-function and APT1 index for North Atlantic marine heatwave annual frequency. Lags range from –14 to 0 years, in which negative lags indicate that the Atlantic Meridional Overturning Circulation (AMOC) leads the APT index. The stipples refer to the regions where correlations are statistically insignificant at the 95% confidence level. The base map is from NCAR Command Language map outline databases.

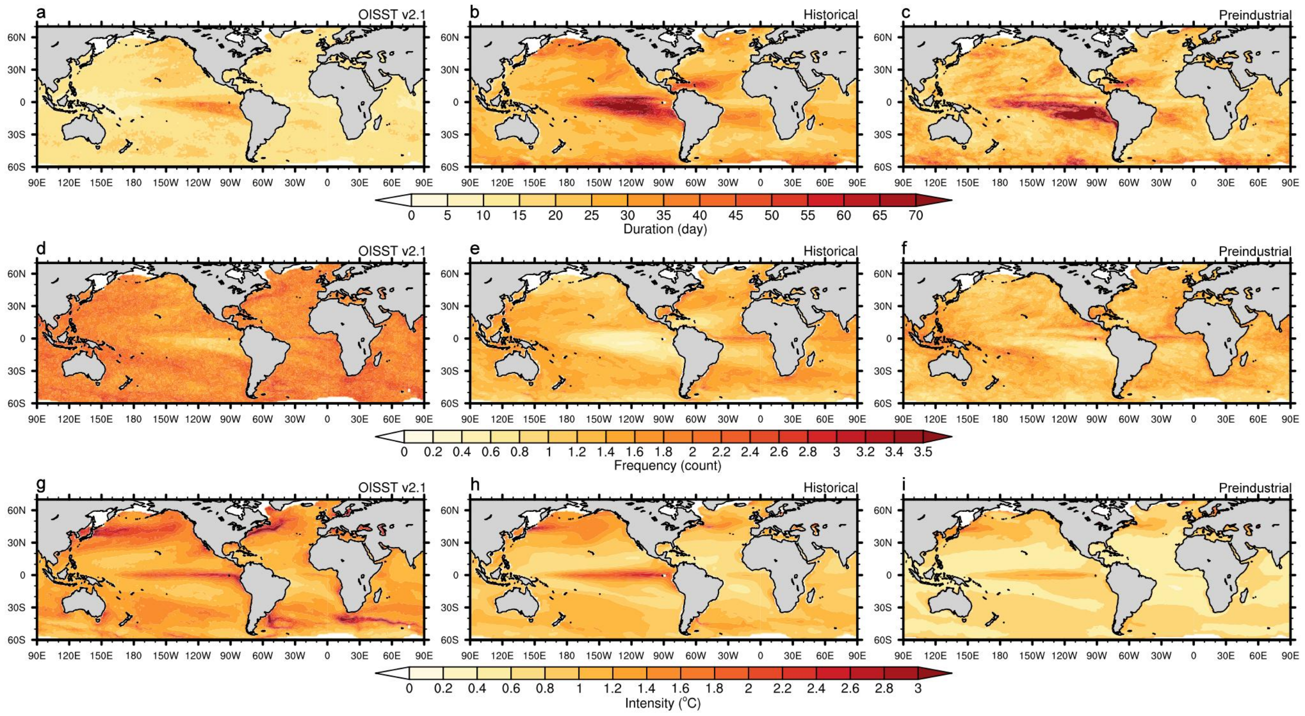

Supplementary Fig. 10 | Observed and simulated global marine heatwaves. (a-c) Marine heatwave durations from (a) OISSTv2.1 and (b) CESM1 historical ensemble mean over 1982-2024, as well as (c) CESM1 preindustrial simulation (i.e., the "dynamic ocean" simulation specified in this study). (**d–f)** Same as (a–c) but for annual mean marine heatwave frequencies. (**g–i)** Same as (a–c) but for marine heatwave intensities. The base map is from NCAR Command Language map outline databases.

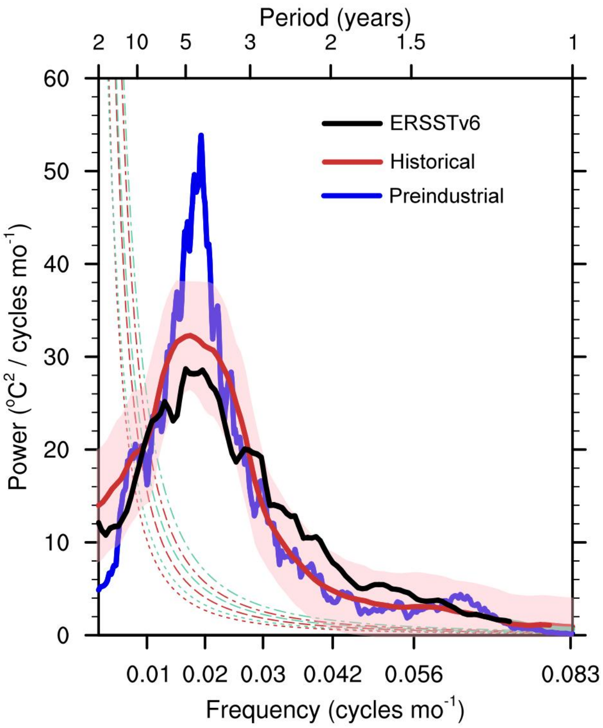

Supplementary Fig. 11 | Observed and simulated El Niño-Southern Oscillation. Power spectra of the Niño 3.4 indices from ERSSTv6 (black) and CESM1 historical large ensemble simulations during 1920-2024 (red, ensemble mean; light red, one standard derivation among ensembles), and CESM1 preindustrial simulation (blue), as well as their 95% confidence limits (dashed/dotted curves).

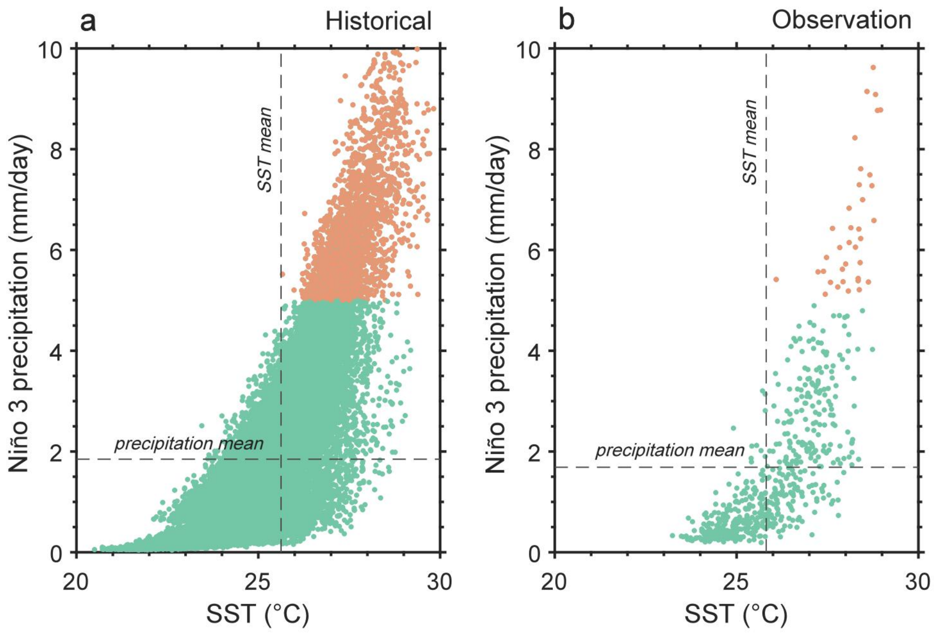

Supplementary Fig. 12 | Observed and simulated extreme El Niño-Southern Oscillation. Scatterplots of monthly sea surface temperature versus monthly precipitation in the Niño 3 region for (**a**) CESM1 historical large ensemble simulation and (**b**) ERSSTv6 and CMAP observations. Orange dots represent cases when precipitation is larger than 5 mm/day. The black vertical and horizontal dashed lines denote the climatological mean values of sea surface temperature and precipitation, respectively.

Supplementary Table

Model	Dynamic ocean (years)	Slab ocean (years)
CGCM3.1(T47)	1001	30
CGCM3.1(T63)	1001	30
CSIRO-Mk3.0	380	60
GFDL-CM2.0	500	50
GFDL-CM2.1	500	100
GISS-ER	500	120
INM-CM3.0	330	60
MIROC3.2 (medres)	500	60
ECHAM5/MPI-OM	506	100
UKMO-HadCM1	240	71

Supplementary Table 1. CMIP3 models and their simulations. CMIP3 models used in this study, and their experiment durations for simulations with dynamic and slab oceans.
